# *Fasciola hepatica* in UK horses

**DOI:** 10.1111/evj.13149

**Published:** 2019-07-21

**Authors:** A. K. Howell, F. Malalana, N. J. Beesley, J. E. Hodgkinson, H. Rhodes, M. Sekiya, D. Archer, H. E. Clough, P. Gilmore, D. J. L. Williams

**Affiliations:** ^1^ Institute of Infection and Global Health University of Liverpool Liverpool UK; ^2^ Institute of Veterinary Science University of Liverpool Liverpool UK; ^3^ School of Veterinary Medicine University College Dublin Dublin Ireland; ^4^ Institute of Psychology, Health and Society University of Liverpool Liverpool UK; ^5^ Liverpool Veterinary Parasitology Diagnostics University of Liverpool Liverpool UK

**Keywords:** horse, *Fasciola hepatica*, ELISA, microsatellite

## Abstract

**Background:**

*Fasciola hepatica* (liver fluke) affects grazing animals including horses but the extent to which it affects UK horses is unknown.

**Objectives:**

To define how liver fluke affects the UK horse population.

**Study design:**

Descriptive, cross‐sectional, observational study.

**Methods:**

An *F. hepatica* excretory‐secretory antibody detection ELISA with a diagnostic sensitivity of 71% and specificity of 97% was validated and used to analyse serum samples. An abattoir study was performed to determine prevalence. A case‐control study of 269 horses compared fluke exposure between horses with liver disease and controls. Data on clinical signs and blood test results were collected for sero‐positive horses. Genotyping of adult fluke was used to produce a multilocus genotype for each parasite.

**Results:**

Four (2.2%) of 183 horses registered in the UK, sampled in the abattoir, had adult flukes in the liver, and the sero‐prevalence of *F. hepatica* was estimated as 8.7%. In the case‐control study, horses showing signs consistent with liver disease had significantly higher odds of testing positive for *F. hepatica* on ELISA than control horses. In 23 sero‐positive horses, a range of non‐specific clinical signs and blood test abnormalities was reported, with a third of the horses showing no signs. Genotypic analysis of liver flukes from horses provided evidence that these came from the same population as flukes from sheep and cattle.

**Main limitations:**

Bias could have arisen in the prevalence and case‐control studies due to convenience sampling methods, in particular the geographic origin of the horses. Only a small number of horses tested positive so the data on clinical signs are limited.

**Conclusions:**

Exposure to liver fluke occurs frequently in horses and may be an under‐recognised cause of liver disease. Flukes isolated from horses are from the same population as those found in ruminants. When designing and implementing parasite control plans, fluke should be considered, and horses should be tested if appropriate.

## Introduction

*Fasciola hepatica* (liver fluke), is a common and widespread pathogen, well‐known for its effects on the health and productivity of ruminants. Horses frequently graze the same pastures as sheep and cattle but are thought to be relatively resistant to liver fluke infection 1, 2. It is thought that fluke infections in horses often do not reach maturity 2 and in those that do, the prepatent period may be longer or eggs may not be excreted in faeces 1, 3, 4. This leads to difficulty in diagnosis using faecal egg detection methods. However, there is evidence from case reports 5, 6, 7, 8 and experimental infections 2, 3, 4 that horses can be adversely affected by *F. hepatica*, with clinical signs including poor performance, fatigue, diarrhoea, inappetence and jaundice. At the same time, liver disease is common in horses and the cause can often be difficult to find 9, 10. These factors together led to a concern that liver fluke in horses may be responsible for some cases of undiagnosed equine liver disease.

The aim of this study was to define how liver fluke affects the UK horse population. Our objectives were to optimise an antibody‐detection ELISA to aid in diagnosis; to determine the prevalence of liver fluke infection in an abattoir population; to compare the genotypes of flukes isolated from horses with those found in British sheep and cattle; and, using a case‐control study, to investigate whether liver fluke could be a cause of equine liver disease.

## Materials and methods

### ELISA validation

An *Fasciola hepatica* excretory secretory (ES) antibody detection ELISA validated in cattle 11 was modified. The positive control was a horse that was positive for fluke eggs on faecal egg count. The negative control was a horse that was kept indoors and was assumed not to have been exposed to *F. hepatica*. A panel of 34 known positive and 65 known negative samples were used to test sensitivity and specificity and to choose a diagnostic cut‐off. A composite reference standard (pseudo gold standard) was used to define the positive animals 12, and horses were considered positive if they had fluke eggs found in their faeces and/or had flukes found in the liver at post‐mortem. The positive samples came either from abattoir submissions or from cases submitted to the University of Liverpool Veterinary Parasitology Diagnostic service. Negative horses were sourced from abattoirs and had a detailed liver examination which was negative for fluke as well as having a negative faecal egg count. The negative samples and some positive samples were kindly donated by University College Dublin (UCD).

The ELISA was performed in a similar way to 11 with minor modifications (Supplementary Item 1). Receiver operating characteristic (ROC) analysis was used to determine the optimum cut‐off value. Ninety‐five per cent confidence intervals were estimated by performing 2000 stratified bootstrap replicates using ROCR package in R 13, 14.

### Abattoir study

Nine visits were made to an abattoir in England, between January and December 2017. On each visit, between 20 and 69 horses were slaughtered. The liver of each animal was externally inspected for liver flukes. Only external inspection was possible because the meat inspection protocol in horses does not include incising the liver. Livers from which flukes were isolated were then dissected along the bile ducts to extract all of the flukes. These were then stored in 70% ethanol. A sample of blood was collected from the heart of each animal for testing using the ES ELISA. Information about the age, breed, sex and origin of each horse was recorded from the passport. Blood and fluke samples were taken to the University of Liverpool for analysis. Descriptive statistics were prepared.

### Genotyping of parasites and population genetic analysis

A total of 123 flukes from seven horses were analysed, which included flukes from four UK horses in the abattoir study, two horses subjected to euthanasia in an abattoir in Ireland, and one post‐mortem case. DNA extraction was performed and genotyping was performed using a previously validated panel of microsatellites 16, 17. A multilocus genotype (MLG) for each parasite was determined and alleles and genotypes identified in flukes from horses were compared with those from sheep and cattle. For full details see Supplementary Item 2.

### Case‐control study

#### Definition and recruitment of cases

A case was defined as a horse that was considered by the treating veterinary surgeon to have clinical signs and/or blood test results consistent with liver disease. Cases were recruited by advertising through social media and the veterinary and equine press. Samples were taken between January 2017 and January 2018. Veterinary surgeons were asked to obtain owners' permission for their contact details to be given to the research team and to send in a blood or serum sample and provide details of clinical signs and diagnostic test results. Study information was provided to owners by post, and following confirmation of consent, owners were asked to fill in a questionnaire (Supplementary Item 3), either online or by telephone, to provide information about the signallment of the animals, region of origin and grazing history. The questionnaire was piloted prior to use. Samples were tested on the ES ELISA as described above, and results were reported back to the veterinary surgeons. Gamma‐glutamyl transferase (GGT) and glutamate dehydrogenase (GLDH) activities were assayed by Langford Vets, University of Bristol.

#### Definition and recruitment of controls

Archived serum from Diagnosteq, the equine parasitology diagnostic service at the University of Liverpool, was used as the control population. All archived serum was residual from submission for the purpose of tapeworm testing, and permission for anonymised use in research had been given by the client. All archived serum was collected between January 2017 and January 2018. For samples submitted to Diagnosteq via the Philip Leverhulme Equine Hospital (PLEH), the age, breed, use, grazing history and clinical reason for admission to the hospital were extracted from clinical records. Horses which were known to have liver disease were excluded. No other information was available for samples submitted by other veterinary practices. The postcode of the submitting practice was used to indicate the approximate origin of the horse. Serum samples were tested on the ES ELISA, and for GGT and GLDH levels. If either was raised, the animal was excluded because increased levels of these enzymes are associated with liver disease.

### Data analysis

Wilson confidence intervals were calculated for the prevalence estimates. Full data for all horses were not available, however, the signallment data were used as far as possible to compare the case and control groups using Wilcoxon and Chi square tests. Maps were used to compare the spatial distribution of the cases and controls. A mixed effects logistic regression model was used to compare liver fluke sero‐positivity between the case and control groups. This was done using the lme4 package 16 in R 14. The outcome variable was case or control, with liver fluke ELISA result (positive/negative) as the fixed effect and yard as a random effect.

#### Descriptive clinical study: seropositive horses

Horses (whether recruited as cases or controls) which were positive for liver fluke on ELISA were identified and further information was gathered via an owner questionnaire and/or extracted from clinical records on signallment, management, clinical signs and biochemistry results. These data were summarised and Wilson confidence intervals were calculated for the prevalence estimates.

## Results

### ELISA validation

ROC analysis showed that the area under the curve was maximised at a cut off of 15 PP and at this cut off the sensitivity was estimated to be 71% (95% CI 56–85%) and specificity 97% (95% CI 92–100). Test sensitivity/specificity at other cut offs are shown in Supplementary Item 4.

**Figure 1:**
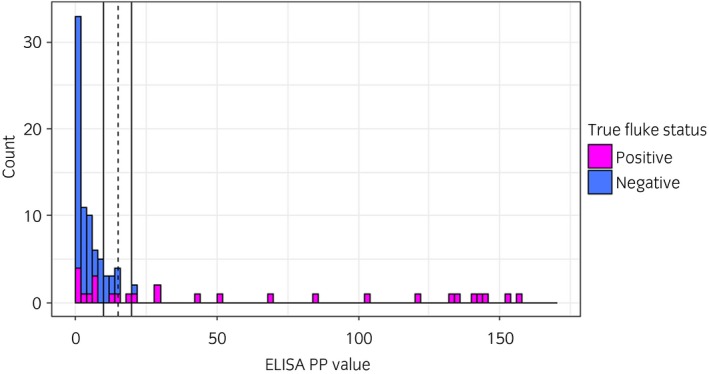
The distribution of *Fasciola hepatica* ES ELISA PP values for horses confirmed positive (by abattoir inspection or faecal egg detection) or negative (by abattoir inspection) for *F. hepatica* infection. The vertical lines indicate PP of 10, 15 (dashed) and 20.

All of the negative horses had a PP of 22 or below, meaning that we can have a high level of confidence in positive results. However, there were several fluke positive horses that failed to mount a detectable antibody response (Fig 1). A cut‐off of 15 PP was used for the rest of the ELISA analysis in this study.

### Abattoir study

**Figure 2:**
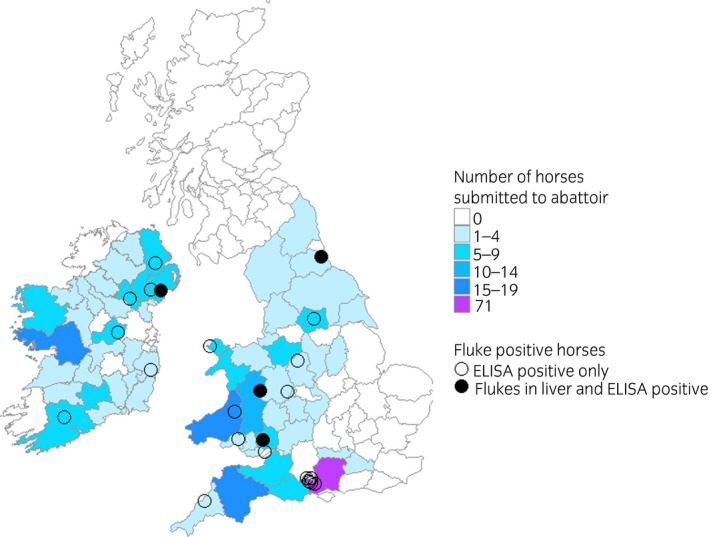
Map showing the most recent origins of the 328 horses from UK and Ireland that were sampled in an abattoir in England, and the origins of the positive horses. The locations have been randomly jittered by 15 km to preserve anonymity.

A total of 342 horses presenting to the abattoir were examined for fluke, of which 224 came from the UK and 104 from Ireland (Fig 2). As a large proportion of the horses were of Irish origin, these data are reported here. Fourteen horses from elsewhere were excluded from further analysis. A range of ages and breeds were represented (Table 1). In total, 9.8% (n = 22) of the UK horses tested positive on ELISA and 1.8% (n = 4) had flukes visible in the liver. Eighteen per cent (n = 41) of the horses were New Forest ponies being culled for population control, and of these, 15% (n = 6) tested positive on ELISA. Excluding the New Forest cull ponies from the overall UK abattoir population gave a fluke‐visual prevalence of 2.2% (n = 4) and a sero‐prevalence of 8.7% (n = 16). The horses with flukes in the liver had 3, 7, 31 and 42 flukes, respectively. Macroscopically, the two horses with the heaviest burdens had visible thickening of the bile ducts, but no other pathological changes were seen in the livers of these infected animals. Of the Irish horses 3.9% (n = 4) tested positive on the ELISA. No Irish horses had flukes found in the liver at post mortem examination.

**Table 1:** Characteristics of horses sampled at an abattoir in England (n = 328)

Characteristic	Data	UK horses (n = 224)	Irish horses (n = 104)
Age (years)	Range	1–27	1–39
	Mean	12.0	11.2
	Median	12	11
	Unknown	56	16
Sex	Female	122 (54.5%)	57 (54.8%)
	Male	97 (43.3%)	31 (29.8%)
	Unknown	5 (2.2%)	16 (15.4%)
Breed	Thoroughbred	20 (8.9%)	36 (34.6%)
	Welsh	53 (23.7%)	0
	Sport horse	11 (4.9%)	42 (40.4%)
	Cob	12 (5.4%)	0
	New forest pony	26 (11.6%)	0
	Cull pony^1^	41 (18.3%)	0
	Irish draught	1 (0.4%)	12 (11.5%)
	Other	21 (9.4%)	6 (5.8%)
	Unknown	39 (17.4%)	8 (7.7%)

1 New forest ponies culled for population control.

### Genotyping of parasites and population genetic analysis

A total of 123 flukes from seven horses were analysed. A complete MLG could not be produced for three parasites so they were excluded from the analysis. The numbers of different alleles and genotypes identified at each locus are shown in Supplementary Item 5. Only six novel alleles were found in this horse fluke population compared with the sheep and cattle population previously described 15, indicating a high degree of similarity between the parasite populations from different definitive hosts. Loci Fh_1, Fh_3, Fh_4, Fh_7, Fh_8 and Fh_14 were excluded due to null alleles. Fh_9 was excluded due to a technical error.

There were three sets of two individual flukes with the same MLG. Two of the pairs were isolated from a single horse, and the third pair from a second horse. The P_sex_ values (the probability of a genotype occurring more than once by chance) for these pairs were 7.31 × 10^−10^, 1.81 × 10^−10^ and 2.78 × 10^−10^. These probabilities are very low indicating that these identical individuals were unlikely to have arisen by chance. They are therefore likely to be clones derived from a single snail intermediate host. Genotypic richness gives a measure of the number of distinct genotypes in a population and therefore indicates how much diversity there is in the population. Here genotypic richness was 0.98, showing the population was genetically diverse i.e. that one parasite was highly likely to have a different MLG to another parasite.

F_ST_ is a measure of genetic differentiation in a population. The F_ST_ between fluke populations in individual horses was 0.056. This is low, indicating that there was little genetic differentiation. Low genetic differentiation means that whilst there were a large number of distinct alleles, there was no structure or pattern in the way that these were combined in individual flukes. This indicates that there were no isolated groups of parasites, and that parasites within the population can breed freely. Combining the genetic data from horses with data from sheep and cattle 15 gave an F_ST_ of 0.0236. Comparisons between horses and sheep and horses and cattle separately gave similar results (Supplementary Item 6). This low F_ST_ suggests a population where genes move freely between the different definitive hosts, i.e. that parasites from sheep and cattle can infect horses and vice versa.

### Case‐control study

A total of 162 samples were submitted as cases between January 2017 and January 2018. Of these, 53 horses did not have any clinical signs or blood biochemistry abnormalities and thus were excluded from the analysis, leaving 109 cases. These were submitted from 49 veterinary practices and from 94 different yards. The maximum number of samples sent from any one practice was 15, and from any one yard was four.

A total of 180 control samples were obtained. Of these, 112 had been submitted from the PLEH and the age, breed, use, grazing history and clinical reason for admission to the hospital were extracted. Horses which were known to have liver disease were excluded. Most of the horses had presented with colic. The other 68 samples had been submitted by veterinary practices and no other information was available for these animals. Twenty horses were excluded due to increased GGT and/or GLDH activity, leaving 160 controls.

**Figure 3:**
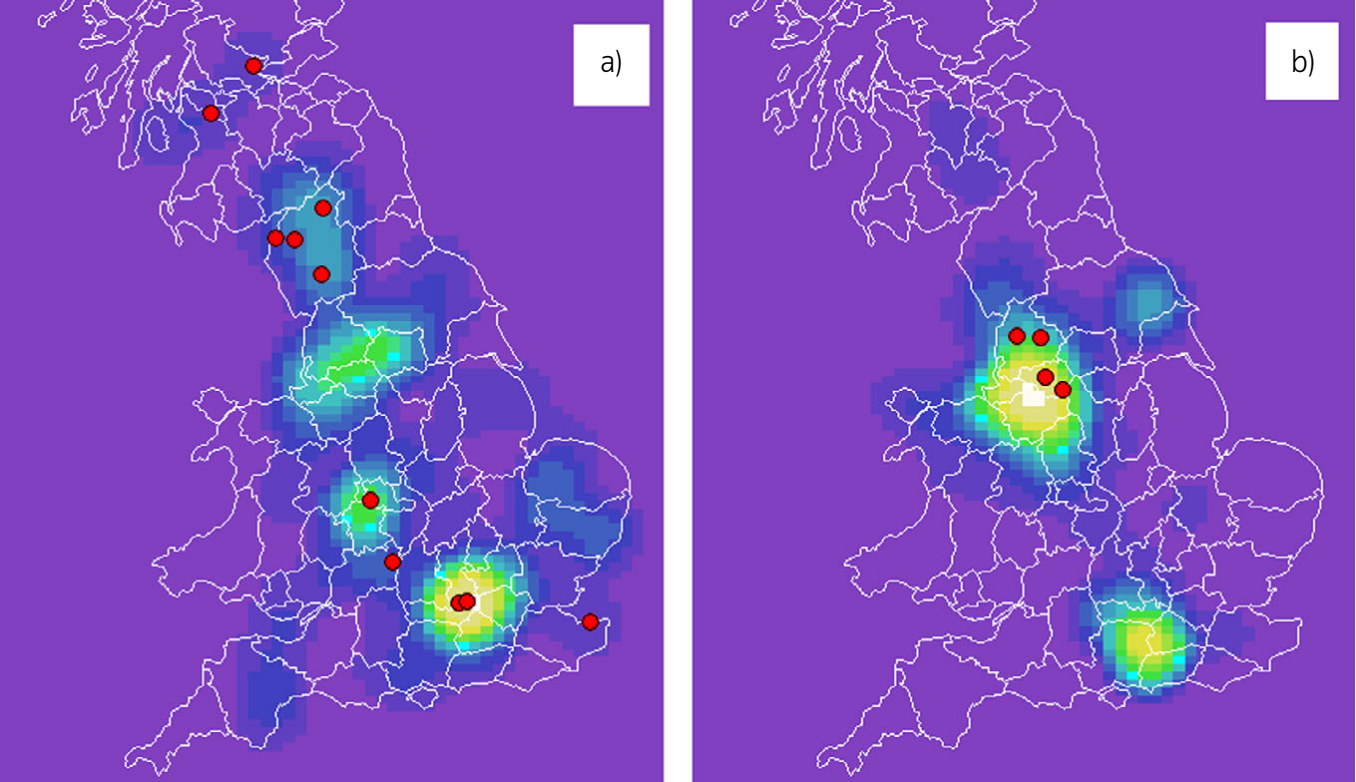
Kernel density plots showing distributions of the cases a) and controls b). The red dots indicate *Fasciola hepatica* ES antibody ELISA positive horses. The locations have been randomly jittered by 15 km to preserve anonymity.

The liver disease cases originated from across the UK, although Wales and Northern Ireland were under‐represented. The controls were clustered around North West England, which reflects the origin of most horses presenting to the PLEH. Horses testing positive for liver fluke originated from various locations (Fig 3).

No management/signalment data were available for approximately one third of the cases and the controls. Analysing the available data, there were no significant differences between the cases and controls in any of the variables except for grazing history (Table 2).

**Table 2:** Characteristics of cases and controls

Variable	Category	Cases (n = 109)	Controls (n = 160)	P value
Age (years)	Range	1–30	0.5–22	0.06
	Mean	12.8	10.8	
	Median	12.0	11.00	
	Unknown	15 (13.8%)	63 (39.4%)	
Sex	Female	39 (39.0%)^1^	39 (40.2%)^1^	>0.9
	Male	61 (61.0%)	58 (59.8%)	
	Unknown	9 (8.3%)	63 (39.4%)	
Breed/type	Thoroughbred	11 (11.5%)	12 (12.6%)	0.3
	Welsh	14 (14.6%)	11 (11.6%)	
	Sport horse	9 (9.4%)	15 (15.8%)	
	Pony	14 (14.6%)	8 (8.4%)	
	Irish draught	4 (4.2%)	6 (6.3%)	
	Cob	24 (25%)	16 (16.8%)	
	Other	12 (12.5%)	10 (10.5%)	
	Unknown	13 (11.9%)	65 (40.6%)	
Use^2^	General riding	45 (61.6%)	49 (54.4%)	0.7
	Eventing	5 (6.8%)	7 (7.8%)	
	Showing	8 (11.0%)	6 (6.7%)	
	Showjumping	8 (11.0%)	7 (7.8%)	
	Dressage	10 (13.7%)	7 (7.8%)	
	Retired	10 (13.7%)	4 (4.4%)	
	Other	12 (16.4%)	12 (13.3%)	
	Unknown	36 (33.0%)	70 (43.8%)	
Grazing	Recent history of grazing	99 (100%)	77 (83.7%)	**<0.001**
	Not grazing	0	15 (16.3%)	
	Unknown	10 (9.2%)	68 (42.5%)	
Co‐grazing	Recent history of co‐grazing with sheep or cattle	30 (41.1%)	11 (25.6%)	0.1
	No co‐grazing	43 (58.9%)	32 (74.4%)	
	Unknown	36 (33.0%)	125 (73.1%)	

1 Percentages have been calculated from the non‐missing total (apart from the unknowns) to aid comparison.

2 Percentages for each use were higher in the cases. Cases were more likely to have more than one use recorded. This was likely an artefact of the method of data collection.

The bold indicates significant value.

### GLDH, GGT and liver fluke status

Seventeen horses tested positive for liver fluke on ES ELISA. This represented 11.0% of the cases and 2.5% of the controls (Table 3). Seventy‐one per cent and 37% of cases had increased GGT and GLDH activities, respectively.

**Table 3:** GLDH, GGT and *Fasciola hepatica* ES ELISA results in the cases and the controls

Variable	Outcome	Cases (n = 109)	Controls (n = 160)
GGT	Raised	71 (71%)	0
	Not tested^1^	9 (8.3%)	0
GLDH	Raised	37 (38.5%)	0
	Not tested^1^	13 (11.9%)	0
ELISA result (cases and controls)	Liver fluke positive	12	4
	Sero‐prevalence	11.0% (95% CI 6.4–18.3%)	2.5% (95% CI 1.0–6.3%)
ELISA result (all horses)^2^	Liver fluke positive	23/342	
	Sero‐prevalence	6.7% (95% CI 4.5–10.0%)	

1 Samples not tested due to insufficient sample left.

2 This includes those horses excluded from the case‐control study due to having no clinical signs (cases) or increased GGT/GLDH (controls).

### Association between liver fluke infection and liver disease

Using the mixed effects logistic regression model and controlling for yard, the odds ratio for exposure to liver fluke was 6.4 (95% CI 1.6–25.7, P = 0.009) for cases compared with controls.

### Descriptive clinical study: seropositive horses

In total 23 horses tested positive for fluke on the ELISA. This included 12 cases, four controls, four in contact horses and three horses excluded from previous analysis due to absence of blood test results. The age range of the positive horses was 5–30 years, with a median of 12. A variety of breeds and types of horse were represented. All positive horses where information was available had a recent grazing history, and 9/14 were known to have grazed with ruminants. This represented 64% (95% CI 35–87%) of the positive horses, compared with 38% (95% Wilson CI 30–47%) of negative horses, a non‐significant difference. Lethargy, dullness and weight loss were the most frequently reported clinical signs, reported in around half of the animals. Inappetence, fever, colic, poor performance, diarrhoea, jaundice and head pressing were less frequently reported. Half of the horses had increased GGT activity whilst a smaller proportion had increased GLDH activity. Additional blood biochemistry results were available for six horses. Of these, none had eosinophilia or anaemia. Raised levels of alkaline phosphatase, aspartate transaminase, bile acids and/or hypoalbuminaemia were observed in a proportion of these six horses.

## Discussion

This is the first study to look at *Fasciola hepatica* in the UK‐wide horse population. We have demonstrated that exposure to liver fluke occurs frequently in horses, and that it may be an under‐recognised cause of liver disease. Moreover, based on the low F_ST _value, we showed that flukes isolated from horses are from the same population as those found in ruminants. Parasite diagnosis and control programmes should therefore include consideration of fluke infection in certain circumstances.

The ES ELISA is currently the most sensitive diagnostic test commercially available for *F. hepatica* in horses. Its specificity of 97% and sensitivity of 71% compare favourably with faecal egg detection using sedimentation or zinc sulphate flotation techniques, which can have a sensitivity as low as 16% 17. Interestingly, our results show that some horses seem to fail to mount a detectable antibody response to ES antigen. Similar findings, using various natural and recombinant antigens, in both naturally and experimentally infected horses have been reported previously 4, 18, 19. This appears to be different to the situation in cattle, where there is a consistent antibody response in the majority of infected animals 11.

In the abattoir study, the sero‐prevalence to ES ELISA was estimated at 8.7%. Meanwhile, the ES ELISA prevalence for horses, which had samples submitted for the case‐control study, was 6.7%. Convenience sampling was used for both studies, which led to differences in the geographic origin of the sampled horses. A higher proportion of horses from Wales and Northern Ireland (both relatively high–fluke areas 20) were included in the abattoir study than the case‐control study. New Forest cull ponies, which appear to be at a high risk for fluke, were also over represented in the abattoir study. This population of ponies is under minimal management and freely grazes an area that includes wet boggy ground and waterbodies 21.

In the case‐control study, horses with signs consistent with liver disease had significantly higher odds of *F. hepatica* seropositivity suggesting that liver fluke could be responsible for some undiagnosed cases of liver disease and should be considered as a differential diagnosis. However, the result should be interpreted with caution, as bias may have arisen due to the non‐blinded nature of the case selection and differences in geographic origin between the cases and the controls. Although we asked veterinary surgeons to send in samples from horses with liver disease of any cause, they were aware that the samples would be tested for liver fluke, which could have affected the choice of horses from which samples were submitted. The spatial distribution of the GB horse population was described by Boden *et  al*. 22 and corresponds reasonably well with the distribution of our liver disease cases, but the method of recruitment for controls led to spatial clustering (Fig 3). Liver fluke is more common in Wales and the west of England due to the wetter climate being more suitable for the intermediate snail host, so geographic origin is an important risk factor 23. Representative sampling of controls remains one of the significant challenges for studies of this nature.

In the fluke positive horses, clinical signs and serum biochemistry results were vague and variable, whilst several horses had no detectable abnormalities. This is consistent with other studies 2, 4, 19, 24, 25. Although some abnormalities would be expected in the serum biochemistry of a liver fluke infected horse, the exact abnormalities would depend on the stage and intensity of infection and thus are likely to be more useful as a prognostic rather than as a diagnostic aid.

The evidence from the genetic analysis supports the hypothesis that flukes found in horses, sheep and cattle come from the same population. Horses, sheep and cattle often share the same grazing, which enables high levels of gene flow between flukes infecting the different species 26, 27, 28 Although the sample size was small, our data suggest that horses that co‐graze with ruminants, on pastures with suitable habitat to sustain the intermediate host, may be at higher risk of liver fluke infection. This is probably because of the higher fluke egg output seen in cattle and sheep compared with horses 25.

This raises the question of whether the recommendation to co‐graze horses with ruminants, as a strategy to reduce nematode egg burden on pasture, is still appropriate. We believe that in most situations, the benefits of co‐grazing for nematode control outweigh the potential risk from liver fluke. The decision of whether to co‐graze should be made after considering the local environment and liver fluke infection history. On dry pastures that do not support *G. truncatula* there is no increased risk of liver fluke from co‐grazing. Furthermore, many horses graze with infected cattle and sheep without showing signs of infection 25. Whilst liver fluke infection in the horse can cause serious and chronic disease, if it is diagnosed early it can be easily and effectively treated 5, 7, 25. Although there is no licensed treatment for horses with liver fluke, use of triclabendazole at 15 mg/kg p/o 28 or closantel at 10 mg/kg p/o 8 are reported in the literature, and UK veterinary surgeons reported their use in a 2017 survey (H. Rhodes, unpublished observations). Other possibilities include oxyclosanide at 10 mg/kg p/o 5 or nitroxynil at 7 mg/kg s/c.

28. With these factors in mind, a pragmatic approach to co‐grazing on fluke‐risk pasture is to remain aware of the potential for liver fluke infection, and to test and/or treat promptly if signs consistent with liver fluke are seen.

In conclusion, this study is the first of its kind in the UK and we demonstrate that the prevalence of liver fluke seroprevalence was 10% in UK horses. Horses with liver disease had significantly higher odds of *F. hepatica* seropositivity (OR 6.4, 95% CI 1.6–25.7), indicating that liver fluke should be included as a diagnostic differential for horses with liver disease, especially those with a history of grazing on pastures used by sheep and cattle.

## Authors' declaration of interests

No competing interests have been declared.

## Ethical animal research

Ethical approval was received from the University of Liverpool's Veterinary Research Ethics Committee (VREC 509 and 559).

## Owner informed consent

Horse owners gave consent for their animals' inclusion in the case‐control study. Owners of horses from which control samples were collected gave consent for research in general.

## Source of funding

Animal Welfare Foundation (AWF).

## Authorship

D. Williams, F. Malalana, J. Hodgkinson, D. Archer and H. Clough designed the study. A. Howell collected and analysed data and wrote the report. N. Beesley assisted with molecular work, analysis and writing the report. N. Beesley, H. Rhodes, M. Sekiya and P. Gilmore contributed to data collection. All authors had intellectual input into the study and gave their final approval of the manuscript.

## Supporting information


**Supplementary Item 1: **ELISA modifications.

**Supplementary Item 2: **Details of genotyping methods.

**Supplementary Item 3: **Questionnaire.

**Supplementary Item 4: **Sensitivity and specificity of the *F. hepatica* ES ELISA at various cut offs, with 95% confidence intervals derived from 2000 stratified bootstrap replicates.

**Supplementary Item 5: **Numbers of unique alleles and genotypes for 123 flukes from horses.

**Supplementary Item 6: **F_ST_ between different population groups.

https://www.podbean.com/eu/pb-uw45t-ca264a
