# Supplementary material for: Fasciola hepatica in UK horses
Source: Equine Vet J. 2019 Jul 21;52(2):194–9. doi: 10.1111/evj.13149 (PMC7027485; doi:10.1111/evj.13149)
Supplement: Supplementary file 1 — Supplementary Item 1: ELISA modifications. [file EVJ-52-194-s001.pdf]

BEVA Equine Veterinary Journal

Supplementary Item 1: ELISA modifications.

The blocking step was done with 2% bovine serum albumin (BSA). BSA was found to block more effectively leading to lower non-specific binding. Secondly, the horse serum was diluted to 1 in 200. Thirdly, a goat anti-horse antibody conjugated to horse radish peroxidase (Bio Rad) was used. A checkerboard titration was performed to determine the correct conjugate dilution, before testing samples. The conjugate dilution was selected to ensure that the optical density (OD) of the positive control fell between 1 and 2. ODs were blank corrected and the percentage positivity (PP) of samples calculated against the positive control.
