# Supplementary material for: Fasciola hepatica in UK horses
Source: Equine Vet J. 2019 Jul 21;52(2):194–9. doi: 10.1111/evj.13149 (PMC7027485; doi:10.1111/evj.13149)
Supplement: Supplementary file 2 — Supplementary Item 2: Details of genotyping methods. [file EVJ-52-194-s002.pdf]

BEVA Equine Veterinary Journal

Supplementary Item 2: Details of genotyping methods.

A total of 123 flukes from seven horses were analysed, which included flukes from four UK horses in the abattoir study, two horses killed in an abattoir in Ireland and one post mortem case. DNA extraction was performed using DNEasy Blood and Tissue Kit (Qiagen). A section from the anterior of each parasite approximately 20 mg in size was used. Genotyping was performed using a previously validated panel of microsatellites [1,2]. A multilocus genotype (MLG) for each parasite was determined using PCR and capillary electrophoresis [1,2] with modifications: a 3500XL genetic analyser was used with Genescan Liz-600 v2.0 (Life Technologies). Data was collected using 3500 Series Data Collection Version 2.

Where a complete MLG could not be determined for a parasite, it was excluded from further analysis. The frequency of alleles and genotypes at each locus was determined using Genepop 4.2.1 [3]. Null alleles are alleles not detected by PCR due to the mutation of binding sites. As subsequent analyses are based on the frequency of each allele in a population, loci with null alleles were excluded from analysis. GenClone 2.0 [4] was used to identify individuals sharing the same MLG. To determine whether these parasites are clones produced following clonal expansion within the snail intermediate host or have occurred by chance, an F_{IS} adjusted p_{sex} value was calculated (this is the probability of a genotype occurring more than once by chance) [5]. GenClone2.0 was then used to calculate genotypic richness, which is a measure of the number of distinct genotypes and thus genetic diversity.

To calculate whether there was any evidence of population structure, or whether genes could move freely in the parasite population, F_{ST} values were calculated using Genepop 4.2.1, with the parasites from each horse considered a sub-population. Parasites with identical MLGs were removed from the dataset as this would skew the data in a similar way to the presence of null alleles. Due to a hardware upgrade, a conversion was required to compare alleles identified in horses and those identified in cattle and sheep from Beesley *et al*. (2007) [2]. Following this conversion an overall F_{ST} value for the fluke from horses and the fluke from sheep and cattle was derived.

References

1. Cwiklinski, K., Allen, K., Lacourse, J., Williams, D.J., Paterson, S. and Hodgkinson, J.E. (2015) Characterisation of a novel panel of polymorphic microsatellite loci for the liver fluke, Fasciola hepatica, using a next generation sequencing approach. *Infect. Genet. Evol.* **32**, 298-304.

2. Beesley, N.J., Williams, D.J.L., Paterson, S. and Hodgkinson, J. (2017) Fasciola hepatica demonstrates high levels of genetic diversity, a lack of population structure and high gene flow: possible implications for drug resistance. *Int. J. Parasitol.* **47**, 11-20.

3. Rousset, F. (2008) genepop'007: a complete re-implementation of the genepop software for Windows and Linux. *Mol. Ecol. Resour.* **8**, 103-106.

4. Arnaud-Haond, S. and Belkhir, K. (2006) Genclone: a computer program to analyse genotypic data, test for clonality and describe spatial clonal organization. *Mol. Ecol. Notes* **7**, 15-17.

5. Parks, J.C. and Werth, C.R. (1993) A study of spatial features of clones in a population of bracken fern, Pteridium aquilinum (dennstaedtiaceae). *Am. J. Bot.* **80**, 537-544.
