# Supplementary material for: Fasciola hepatica in UK horses
Source: Equine Vet J. 2019 Jul 21;52(2):194–9. doi: 10.1111/evj.13149 (PMC7027485; doi:10.1111/evj.13149)
Supplement: Supplementary file 3 — Supplementary Item 3: Questionnaire. [file EVJ-52-194-s003.pdf]

BEVA Equine Veterinary Journal

**Supplementary Item 3:** Questionnaire.

**The impact of liver fluke infection on the welfare of horses: telephone questionnaire**

## Section 1: About your horse

1. What age is the horse?...............................................
2. What breed?.............................................................
3. Male or female?.........................................................
4. What is the origin of your horse? (eg. region of UK or other country)

   …………………………………………………………………………….
5. Where is the horse currently kept (town/village name)?

   …………………………………………………………………………………….
6. How long has it been kept there?

   …………………………………………………………………………………
7. What is the horse used for? (Circle below)

   Competing (affiliated/unaffiliated

   Eventing, show jumping, dressage, other)

   General riding/hacking

   Field companion

   Retired

   Other…………………………………..

## Section 2: About management of your horse

8. Has your horse been grazing over the past year?

   Yes ☐

   No ☐

   IF YES:

   a. For which and how many months?.............................................................
   b. Is it out:

24/7 ☐

Stabled at night ☐

c. Have sheep, cattle, llamas or donkeys been grazing on this pasture?

…………………………………………………………………………………………………….

d. Are there other animals grazing in surrounding fields?

Yes ☐ If yes, what?......................................................

No ☐

e. Is the grazing dry or boggy, or are there any boggy areas?

Yes ☐ If yes, give details......................................................

No ☐

f. Is there a pond, ditch or stream in the field?

…………………………………………………………………

9. Have there been any changes in management in the last 5 years (eg. new owner or moved location)
…………………………………………………………………………………………………………
…………………………………………………………………………………………………………

## Section 3: About your horse's health

10. Is your horse in good health at the moment?

Yes ☐

No ☐

a. (If no) What signs of being unwell is your horse showing?

………………………………………………………………………………………

b. (If no) How long has your horse been unwell?

…………………………………………………………………………………………

11. Has your horse had a previous diagnosis of liver fluke?

Yes ☐

No ☐

(If yes) Was any treatment given?

Yes ☐ If yes, what?.........................................................

No ☐
