# Supplementary material for: Fasciola hepatica in UK horses
Source: Equine Vet J. 2019 Jul 21;52(2):194–9. doi: 10.1111/evj.13149 (PMC7027485; doi:10.1111/evj.13149)
Supplement: Supplementary file 4 — Supplementary Item 4: Sensitivity and specificity of the F. hepatica ES ELISA at various cut offs, with 95% confidence intervals derived from 2000 stratified bootstrap replicates. [file EVJ-52-194-s004.pdf]

BEVA Equine Veterinary Journal

Supplementary Item 4: Sensitivity and specificity of the *F. hepatica* ES ELISA at various cut offs, with 95% confidence intervals derived from 2000 stratified bootstrap replicates.

**Cut off**	**Sensitivity (95% CI)**	**Specificity (95% CI)**
3.5	88.2% (76.5-97)	55.4% (43.1-67.7)
6.5	82.4% (70.6-94.1)	73.9% (63.1-84.6)
12.5	73.5% (58.5-85.3)	90.8% (83.1-96.9)
15.0	70.6% (55.9-85.3)	96.9% (92.3-1.00)
18.0	67.7% (52.9-82.4)	98.5% (95.4-1.00)
25.5	61.8% (44.1-76.5)	1.00% (1.00-1.00)
