# Supplementary material for: Fasciola hepatica in UK horses
Source: Equine Vet J. 2019 Jul 21;52(2):194–9. doi: 10.1111/evj.13149 (PMC7027485; doi:10.1111/evj.13149)
Supplement: Supplementary file 5 — Supplementary Item 5: Numbers of unique alleles and genotypes for 123 flukes from horses. [file EVJ-52-194-s005.pdf]

BEVA Equine Veterinary Journal

Supplementary Item 5. Numbers of unique alleles and genotypes for 123 flukes from horses**.**

Locus	Nr of alleles	Nr of genotypes
Fh_1*	6	11
Fh_2	14	36
Fh_3*	6	9
Fh_4*	14	42
Fh_5	18	43
Fh_6	19	55
Fh_7*	7	13
Fh_8*	10	26
Fh_9^	2	2
Fh_10	11	33
Fh_11	9	26
Fh_12	12	29
Fh_13	9	13
Fh_14*	12	29
Fh_15	5	8

* excluded due to presence of null alleles, ^ excluded due to technical error
