# Supplementary material for: Fasciola hepatica in UK horses
Source: Equine Vet J. 2019 Jul 21;52(2):194–9. doi: 10.1111/evj.13149 (PMC7027485; doi:10.1111/evj.13149)
Supplement: Supplementary file 6 — Supplementary Item 6: FST between different population groups. [file EVJ-52-194-s006.pdf]

BEVA Equine Veterinary Journal

Supplementary Item 6: F_{ST} between different population groups.

Population groups	F_{ST}
Cattle and sheep	0.0202
Cattle and horse	0.0284
Sheep and horse	0.0247
Cattle, sheep and horse	0.0236
